# Selective Adsorption of Ag^+^ on a New Cyanuric-Thiosemicarbazide Chelating Resin with High Capacity from Acid Solutions

**DOI:** 10.3390/polym9110568

**Published:** 2017-11-02

**Authors:** Guo Lin, Shixing Wang, Libo Zhang, Tu Hu, Jinhui Peng, Song Cheng, Likang Fu

**Affiliations:** 1 State Key Laboratory of Complex Nonferrous Metal Resources Clean Utilization, Kunming University of Science and Technology, Kunming 650093, China; glin08@126.com (G.L.); hutu1219@126.com (T.H.); jhpeng@kmust.edu.cn (J.P.); cs1246@126.com (S.C.); fulk324@163.com (L.F.); 2 Faculty of Metallurgical and Energy Engineering, Kunming University of Science and Technology, Kunming 650093, China; 3 Key Laboratory of Unconventional Metallurgy, Ministry of Education, Kunming 650093, China; 4 National Local Joint Laboratory of Engineering Application of Microwave Energy and Equipment Technology, Kunming 650093, China

**Keywords:** chelating resin, silver ions, selectivity, adsorption, mechanism

## Abstract

A new cyanuric-thiosemicarbazid (TSC-CC) chelating resin was synthesized and employed to selectively adsorb Ag^+^ from acid solutions. The effects of acid concentration, initial concentration of Ag^+^, contact time and coexisting ions were investigated. The optimal acid concentration was 0.5 mol/L. The adsorption capacity of Ag^+^ reached 872.63 mg/g at acid concentration of 0.5 mol/L. The adsorption isotherm was fitted well with the Langmuir isotherm model and the kinetic data preferably followed the pseudo-second order model. The chelating resin showed a good selectivity for the Ag^+^ adsorption from acid solutions. Fourier transform infrared (FT-IR), X-ray diffraction (XRD), Scanning electron microscopy/energy dispersive spectrometer (SEM-EDS) and X-ray photoelectron spectroscopy (XPS) were used to study the adsorption mechanism. The chelating and ionic interaction was mainly adsorption mechanism. The adsorbent presents a great potential in selective recovery Ag^+^ from acid solutions due to the advantage of high adsorption capacity and adapting strongly acidic condition. The recyclability indicated that the (TSC-CC) resin had a good stability and can be recycled as a promising agent for removal of Ag^+^.

## 1. Introduction

The wastewater containing Ag, Hg, Pb, Co, Cd, Ni, and Cu has been more and more noticed because of the potential hazards to human, animals, and the environment [1]. Within these metals, silver is a typical toxic heavy metal coming from coinage, metallurgy, electronics industries, and photography [2,3,4]. The wastewater which silver content exceeds the normal range will cause a series of adverse impact to human and ecological system such as cancer, heart failure, and genovariation on account of the toxicity to the liver, heart, and lung [5]. Therefore, it is necessary to develop effective methods for treating the wastewater and recovering silver.

The traditional methods for recovery Ag^+^ from wastewater include precipitation, electrolysis, ion exchange, membrane separation, and adsorption [3]. Among the above methods, adsorption is an emerging technique due to easy operation, low energy consumption and high-efficiency [6]. A number of adsorbents have been developed for separation and recovery of Ag^+^ such as the modified silica nanoparticle with sulfoethyl groups [7], activated carbon [8], waste coffee grounds [5], and chelating resins [9]. The chelating resins have stood out from the other adsorbents due to their high uptake capacity, high selectivity, and possible modification of their physical and chemical properties compared with activated carbon and ion exchange resins.

Wang et al. [9] separated Ag^+^ from aqueous solution by trimercaptotriazine-functionalized polystyrene chelating resin. They found that the maximum adsorption capacity of Ag^+^ was 187.1 mg/g at pH 0.0 and decreased with pH increasing. Atia et al. [10] anchored different chelating moieties on glycidyl methacrylate/divinylbenzene resin in order to adsorb Ag^+^. The highest uptake of Ag^+^ on glycidyl methacrylate/divinylbenzene resin was 308.51 mg/g at natural pH. However, the uptake decreased obviously in the acidic media. Yirikoglu et al. [11] removed Ag^+^ via melamine-formaldehyde-thiourea (MFT) chelating resin. The highest uptake of Ag^+^ on MFT occurred at high pH values. In chelating resins, sulfur (e.g., S presents in thiols, thiocarbamates, thioethers) and nitrogen (e.g., N presents in amines and amides) are used as donor atoms that interact directly with Ag^+^. The protonation of the donor atoms of adsorbent increases with decreasing pH, resulting in the uptake of Ag^+^ on the chelating resins changes. However, the high uptake of Ag^+^ is especially useful for the practical application even at low pH values. Thus, it is necessary to develop a resin which can absorb the Ag^+^ from acidic solution.

In the paper, a new chelating resin was synthesized via the reaction between cyanuric chloride and thiosemicarbazide. It had the advantages of adsorbing Ag^+^ from acid solutions, high adsorption capacity, and superior selectivity. In addition, the recyclability of the obtained resin for Ag^+^ from aqueous solution was investigated. The effects of acid concentration, initial Ag^+^ concentration, contact time, and interfering ions (Hg, Ni, Mn, Co, Cu, and Zn) on the adsorption performance were examined. Meanwhile, the adsorption kinetics, adsorption isotherms and adsorption mechanism were also studied. The present research may be useful to separate and recover Ag^+^ from wastewater efficiently.

## 2. Experimental

### 2.1. Materials

Sodium carbonate was obtained from Tianjin Ruijinte Chemistry Co., Ltd. (Tianjin, China). The thiosemicarbazide was purchased from Shanghai Macklin Biochemical Co., Ltd. (Shanghai, China), and cyanuric chloride was employed from Aladdin Instruments Corporation (Shanghai, China). All chemicals were analytical grade and used without further treatment. The metal ions standard stock solutions (1000 mg/L) were prepared by adding appropriate amount of nitrate salts (AgNO_3_, Hg(NO_3_)_2_, Ni(NO_3_)_2_, Co(NO_3_)_2_, Cu(NO_3_)_2_, Mg(NO_3_)_2_, and Zn(NO_3_)_2_) to deionized water. Before experiments, all solutions were adjusted with 0.1 mol/L HNO_3_ or 0.1 mol/L NaOH solutions to obtain the required acidity and pH.

### 2.2. Methods

#### 2.2.1. Synthesis of Chelating Resin

**Scheme 1:**
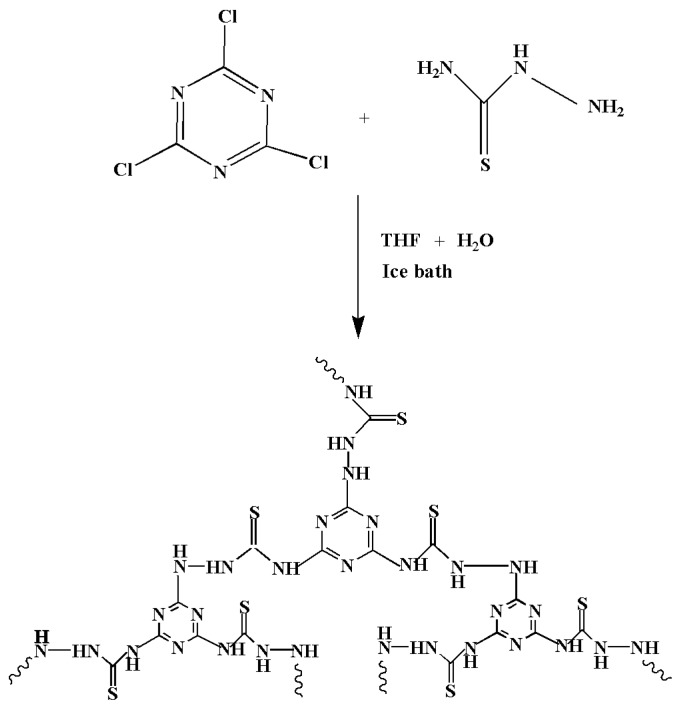
The synthesized process of cyanuric-thiosemicarbazide chelating resin.

Chelating resin was prepared as following (Scheme 1): sodium carbonate (5 g) and thiosemicarbazide (2.8 g, denoted as TSC) were dissolved in 100 mL of Tetrahydrofuran (THF) and 100 mL of deionized water. The experiments were carried out in a 500 mL three-necked flask under an ice bath. Cyanuric chloride (5.53 g, denoted as CC) was dissolved in 40 mL of deionized water and dropped to the three-necked flask. Then the solution was stirred for 10 h. After centrifuged, the obtained solid was washed three times using THF and deionized water, respectively. The solid was dried at 65 °C for 24 h under vacuum and defined as TSC-CC.

#### 2.2.2. Adsorption Experiments

To investigate the adsorption capacity of TSC-CC for silver from aqueous solutions, batch experiments were carried out by mixed silver solutions of 20 mL with the adsorbent of 10 mg in 50 mL centrifugal tube. The centrifugal tubes were placed in a thermostat steam bath vibrator (ZD-85, Jintan experimental instrument, Jintan, China) and shaken at a speed of 300 rpm for 24 h at room temperature.

The effects of acid concentration on recovery of Ag^+^ were performed at the initial Ag^+^ concentration of 300 mg/L at room temperature with HNO_3_ concentration of 1 and 0.5 mol/L, pH 1–6. The experiments of adsorption kinetics were conducted at adsorption time of 5–720 min, initial Ag^+^ concentration of 400 mg/L and acidity of 0.5 mol/L under room temperature. Experiments of adsorption isotherm were carried out at initial Ag^+^ concentrations of 340–800 mg/L and acidity of 0.5 mol/L at room temperature. The selectivity of the adsorbent toward Hg^2+^, Ni^2+^, Co^2+^, Zn^2+^, Mn^2+^ and Cu^2+^ was investigated. 10 mg of TSC-CC was added into 20 mL of solutions containing Ag^+^ (400 mg/L) and coexisting ions (400 mg/L) at acidity of 0.5 mol/L, and then the mixture solutions were shaken for 10 h. After adsorption, TSC-CC was separated from solution by centrifuge and the supernatant was collected and analyzed by inductively coupled plasma atomic emission spectrometry (ICP-AES, Prodigy7, Leeman, Hudson, NH, USA). All the adsorption experiments were implemented for three times.

The removal efficiency (*R*%) and adsorption capacity (*q*_e_ mg/g) of Ag^+^ were calculated as following Equations (1) and (2):

(1) $$R=\frac{\left(C_{0}-C_{e}\right)}{C_{0}}\times 100\%$$

(2) $$q_{e}=\frac{\left(C_{0}-C_{e}\right)}{W}\times V$$

where *C*_0_ and *C*_e_ (mg/L) were the concentration of Ag^+^ solution before and after adsorption, respectively. *V* (L) represented the volume of Ag^+^ solution and *W* (mg) was the mass of TSC-CC.

### 2.3. Analysis

The FT-IR spectroscopy was obtained by Nicolet iS10 (ThermoFisher, Waltham, MA, USA) with a resolution of 4 cm^−1^ to qualitatively identify the chemical function groups of materials. SEM-EDS analysis was detected by XL30 ESEM-TMP (Philips-FEI, Eindhoven, The Netherlands). XRD was performed by X’Pert^3^ Powder (PANalytical, Almelo, The Netherlands) and analyzed by X’Pert HighScore 3.0 (Royal Dutch Philips Electronics Ltd., Amsterdam, The Netherlands). X-ray photoelectron spectroscopy (XPS) was measured with PHI 5000 Versaprobe-II (Physical Electronics, Inc., Chanhassen, MN, USA) using 200 W Mg radiations to determine the surface chemical composition. The concentrations of metal ions (Ag^+^, Hg^2+^, Ni^2+^, Co^2+^, Zn^2+^, Mn^2+^ and Cu^2+^) were tested by ICP-AES (Prodigy7, Leeman, USA). The detection uncertainty of ICP-AES was 1.7%.

## 3. Results and Discussion

### 3.1. Characterization of Cyanuric-Thiosemicarbazide Chelating Resin

**Figure 1:**
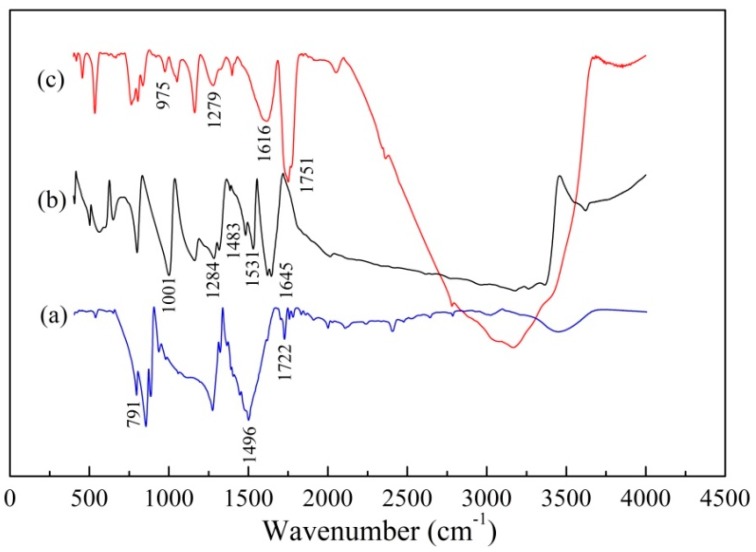
FT-IR spectra of cyanuric chloride (**a**); thiosemicarbazide (**b**) and TSC-CC (**c**).

Figure 1 showed the FT-IR spectroscopy of thiosemicarbazide, cyanuric chloride, and cyanuric-thiosemicarbazide resin. The adsorption band at 2500–3600 cm^−1^ was broad and assigned to the –NH_2_ stretching vibration. For cyanuric chloride, the peaks at 791, 1496, and 1722 cm^−1^ were assigned to the C–Cl, C–N, and C=N stretching vibration [12,13]. In the FT-IR spectrum of thiosemicarbazide, the characteristic adsorption peaks at 1001, 1483, and 1531 cm^−1^ were attributed to C=S, C–N, and N–H stretching and bending vibration [13,14]. The intensity of the bands at 1284 and 1645 cm^−1^ were caused by the –N–C=S group [14]. For TSC-CC, the stretching vibration bands of C=S was observed at 975 cm^−1^ and the peaks at 1616 and 1279 cm^−1^ were the –N–C=S group, respectively. In addition, the peaks at 1751 cm^−1^ was attributed to the stretching vibration of C=N. The results indicated that the chelating resin had been successfully synthesized.

### 3.2. Effect of Initial Acidity on Adsorption

**Figure 2:**
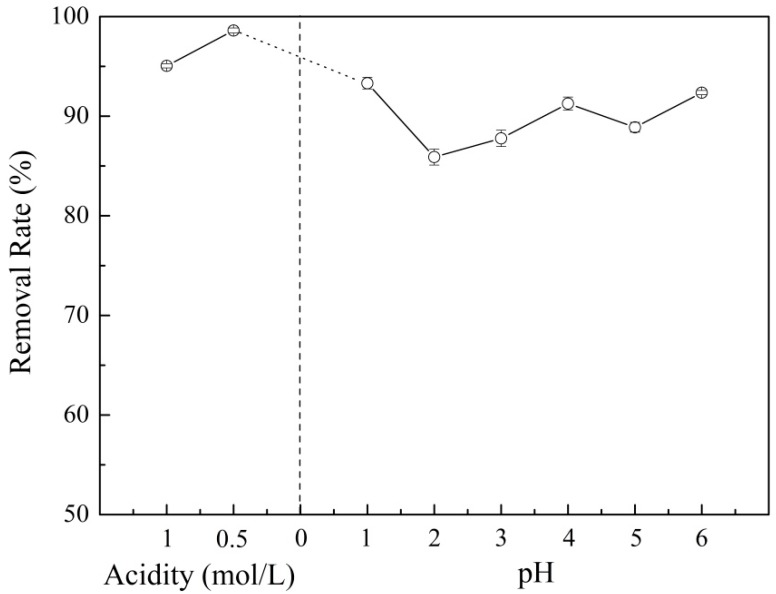
Effect of initial acidity on the removal rate of Ag^+^.

As we all know, the acidity in aqueous solution was regarded as an important parameter because it significantly affected the adsorption efficiency of metal ions by reacting with the active functional groups of adsorbents [15]. To prevent the hydrolysis of Ag^+^, the HNO_3_ concentration of aqueous solution was 1 and 0.5 mol/L, pH 1–6. Figure 2 presented the removal efficiency of Ag^+^ at different initial acidity. The removal rate of Ag^+^ was more than 85% within the scope of acidity. The maximum removal rate was 98.6% when the acidity was 0.5 mol/L, which exceeded the literature reported [16]. Therefore, the optimal acidity was 0.5 mol/L. It indicated that the TSC-CC had a strong adaptability for the aqueous solution and could be employed under different acidity.

Under the acidic solution, Ag^+^ may interact with (R_1_R_2_)NH_2_^+^ functional group by with (N), (S) donor atoms chelation or ionic interaction, and form Ag^+^_(aq)_ (major species) and $\mathrm{Ag}{\left({\mathrm{NO}}_{3}\right)}_{2\left(\mathrm{aq}\right)}^{-}$ (minor species) [17]. The chelation and ionic reactions may be presented as follows equations [10,17,18]:

Chelation reactions:

(3) $$\left(R_{1}R_{2}{\mathrm{NH}}_{2\left(s\right)}^{+}\right)+{\mathrm{Ag}}_{\left(\mathrm{aq}\right)}^{+}=\left(R_{1}R_{2}\right)\mathrm{HN}\ldots {\mathrm{Ag}}_{\left(s\right)}^{+}+H_{\left(\mathrm{aq}\right)}^{+}$$

(4) $$\left(R_{1}R_{2}\right)C=S_{\left(s\right)}+{\mathrm{Ag}}_{\left(\mathrm{aq}\right)}^{+}=\left(R_{1}R_{2}\right)C=S\ldots {\mathrm{Ag}}_{\left(s\right)}^{+}.$$

Ionic reactions:

(5) $$\left(R_{3}\right)NH_{2}^{+}NO_{3(s)}^{-}+\mathrm{Ag}{\left(NO_{3}\right)}_{2(\mathrm{aq})}^{-}=\left(R_{3}\right)NH_{2}^{+}\mathrm{Ag}{\left(NO_{3}\right)}_{2(s)}^{-}+NO_{3(\mathrm{aq})}^{-}.$$

Combined with Equations (3) and (4) and Figure 2, it can be seen that the sulfur atoms had a positive effect for the Ag^+^ adsorption by chelation reactions under pH 2–6. Therefore, it showed that removal of Ag^+^ onto TSC-CC resin was motivated through more ionic interaction due to the adsorption was proceed in acidic solution which the resin can provide more protonation.

### 3.3. Effect of Contact Time on Adsorption and Adsorption Kinetics

**Figure 3:**
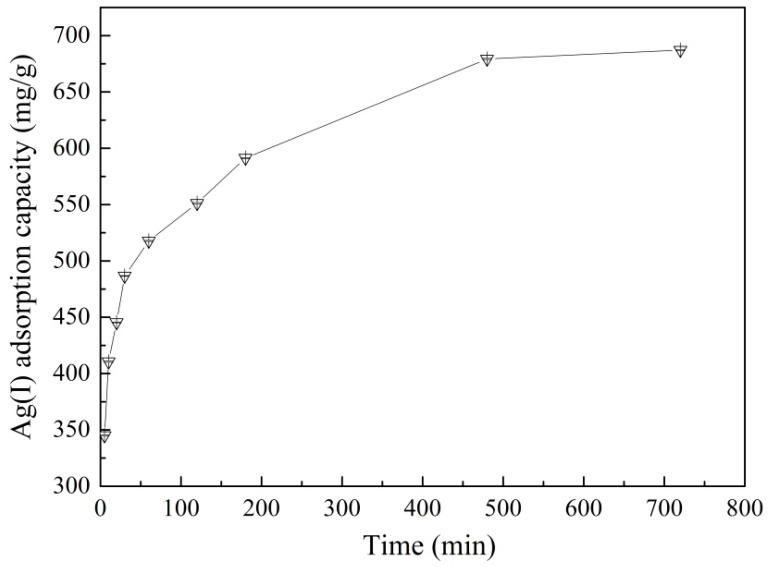
Effect of contact time on the adsorption of Ag^+^ onto TSC-CC resin.

The influence of contact time for the Ag^+^ adsorption onto TSC-CC resin was investigated and the results were showed in Figure 3. The Ag^+^ adsorption capacity rapidly increased before the initial 100 min, and then the saturation and equilibration were nearly obtained after about 500 min. The saturated adsorption capacity of Ag^+^ onto TSC-CC resin was 687.16 mg/g with the Ag^+^ initial concentration of 400 mg/L and acidity of 0.5 mol/L.

During the adsorption process, the adsorption kinetics were described as the removal rate of solute, and it determined the sorbate residence time on the solid-liquid surface. Furthermore, the residence time was regarded as an significant parameter to estimate the adsorption capacity and equilibrium during the adsorption process [19,20]. To research the adsorption mechanism and realize the potential rate-controlling steps such as chemical reaction and mass transport process, the pseudo-first-order, pseudo-second-order, and intraparticle diffusion kinetic models were employed to fit the experimental results. The pseudo-first-order and pseudo-second-order based on solid liquid phase adsorption capacity generally appeared as follows Equations (6) and (7) [19,21]:

(6) $$\ln\left(q_{e}-q_{t}\right)=\ln q_{e}-k_{1}t$$

(7) $$\frac{t}{q_{t}}=\frac{1}{k_{2}q_{e}^{2}}+\frac{t}{q_{e}}$$

and the Equation (6) can be converted to the following equation:

(8) $$q_{t}=q_{e}\left(1-e^{-k_{1}t}\right)$$

where $q_{e}$ (mg/g) was the equilibrium adsorption capacity; $q_{t}$ (mg/g) was the uptake capacity at time t (min); $k_{1}$ (1/min) and $k_{2}$ (g/mg min) were the adsorption rate constant for pseudo-first-order and pseudo-second-order, respectively; *t* (min) was the contact time.

Moreover, the intraparticle diffusion kinetic model was also adopted to investigate the adsorption mechanism and can be showed as follow [22]:

(9) $$q_{t}=k_{3}\sqrt{t}+C$$

where $k_{3}$ (mg/g min^0.5^) was the intraparticle diffusion rate constant.

**Figure 4:**
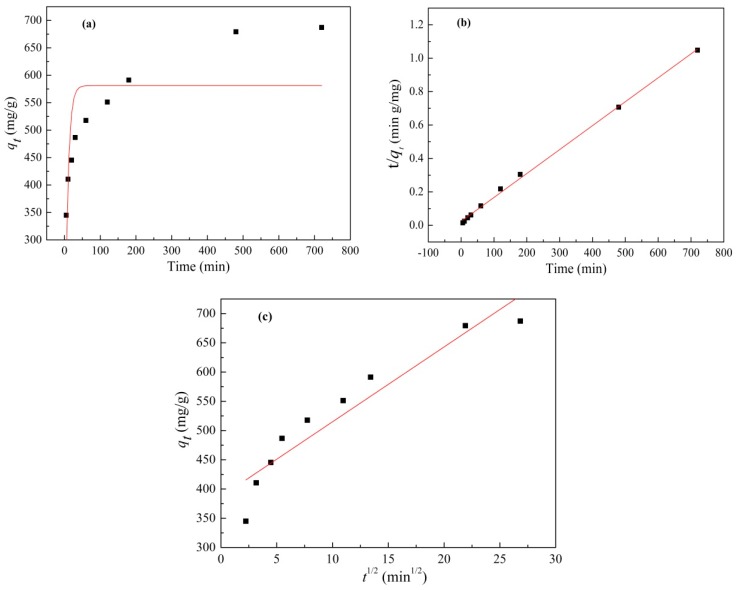
Adsorption kinetics of Ag^+^ on TSC-CC resin, (**a**) pseudo-first-order model; (**b**) pseudo-second-order model; (**c**) intraparticle diffusion model.

Figure 4 showed the kinetics datas and fitting effects, and the obtained parameters were listed in Table 1. The results indicated that pseudo-second-order moder was more appropriate for fitting the actual values compare with pseudo-first-order and intraparticle diffusion kinetic models based on *R*^2^. It can be demonstrated that chemical reaction was the rate-controlling steps during the adsorption process of Ag^+^ onto TSC-CC resin [20,23]. Meanwhile, the lower *R*^2^ for intraparticle diffusion model suggested that the adsorption of Ag^+^ was not controlled by intraparticle diffusion.

### 3.4. Effect of Initial Ag^+^ Concentration on Adsorption and Adsorption Isotherms

**Figure 5:**
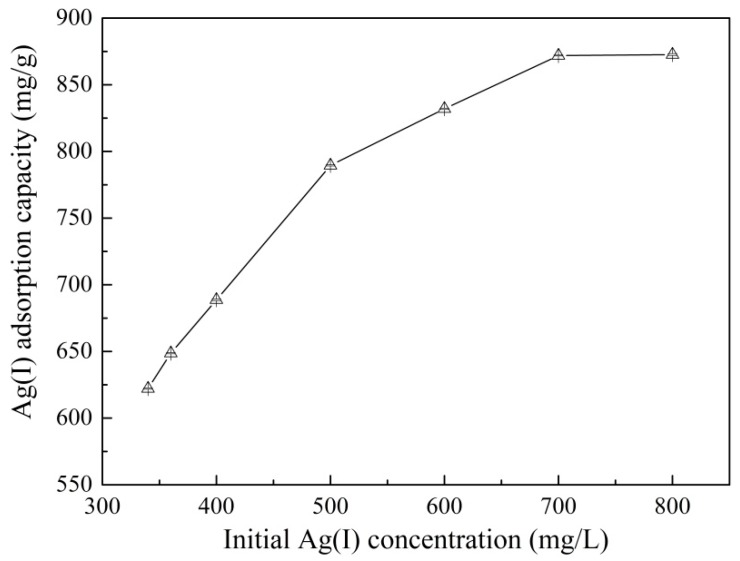
Effect of initial Ag^+^ concentration on the adsorption.

To investigate the maximum adsorption capacity of Ag^+^, the effect of initial Ag^+^ concentration was studied (Figure 5). The adsorption capacity of Ag^+^ increased with initial Ag^+^ concentration up to 700 mg/L. Beyond the concentration, change can hardly be found because the available active sites for interaction with Ag^+^ on the TSC-CC resin reached saturation. The maximum adsorption capacity of Ag^+^ was 872.63 mg/g. The results demonstrated that TSC-CC resin was a promising adsorbent and had large adsorption capacity for Ag^+^. The adsorption capacity of TSC-CC resin and other results reported in literatures were presented in Table 2. As can be seen, TSC-CC resin revealed great potential in removal and recovery of Ag^+^.

In order to investigate the interaction between sorbent and metal ions and comprehend the variation with adsorption capacity and Ag^+^ concentrations, adsorption isotherms were widely employed in the field of metal ions adsorption. Langmuir, Freundlich, and Temkin isotherm models were applied to depict the relationship of adsorption capacity and Ag^+^ concentrations, and further to illuminate the adsorption mechanism of Ag^+^ onto TSC-CC resin. The Langmuir, Freundlich, and Temkin equations were presented as follows, respectively [19,26]:

(10) $$\frac{1}{q_{e}}=\frac{1}{k_{L}q_{m}C_{e}}+\frac{1}{q_{m}}$$

(11) $$\ln\left(q_{e}\right)=\ln\left(k_{F}\right)+\frac{1}{n}\ln\left(C_{e}\right)$$

(12) $$q_{e}=B_{T}\ln\left(k_{T}\right)+B_{T}\ln\left(C_{e}\right)$$

and the Equation (10) can be converted to the following equation:

(13) $$q_{e}=\frac{k_{L}q_{m}C_{e}}{1+k_{L}C_{e}}$$

where $q_{e}$ and $q_{m}$ (mg/g) were the equilibrium and maximal adsorption capacity of Ag^+^, respectively; $C_{e}$ (mg/L) was the equilibrium concentration of Ag^+^; $k_{L}$ (L/g) and $k_{F}$ (mg·g^−1^ (L·mg^−1^)^1/n^) were the Langmuir and Freundlich constant, respectively; *n* was the adsorption intensity constant; $k_{T}$ (L/g) was the binding equilibrium constant and $B_{T}$ (J/mol) was concerned with the adsorption heat.

**Figure 6:**
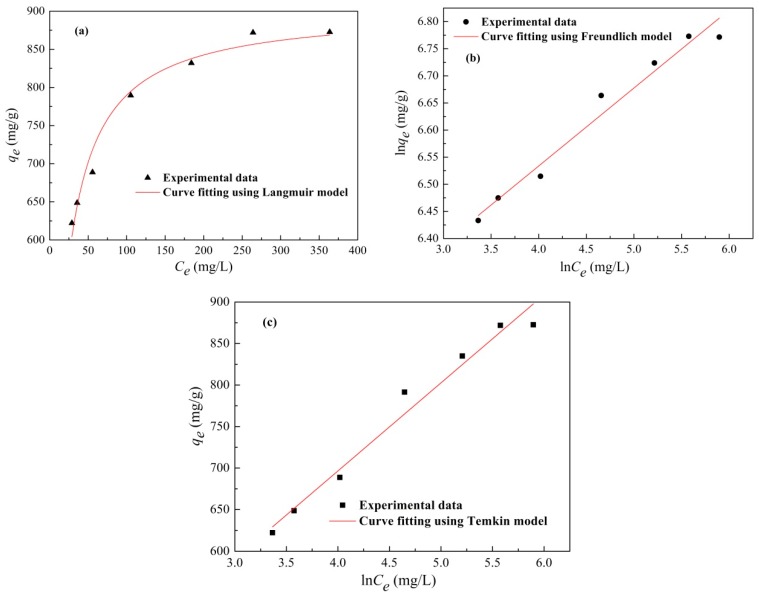
Adsorption isotherms of Ag^+^ on TSC-CC resin, (**a**) Langmuir model; (**b**) Freundlich model; (**c**) Temkin model.

Figure 6 showed the adsorption datas and fitting effects, and the obtained parameters were listed in Table 3. It can be found that the Langmuir isotherm model was more accurate for fitting the experimental data with higher *R*^2^ than Freundlich and Temkin isotherm models. Which indicated that the adsorption process for Ag^+^ onto TSC-CC resin was homogeneous monolayer adsorption on the adsorbent surface [27].

In addition, another important parameter of Langmuir model can be employed to estimate the feasibility of adsorption on adsorbent and was defined as dimensionless factor *R*_L_, it was expressed as follow:

(14) $$R_{L}=\frac{1}{1+k_{L}C_{0}}$$

where $C_{0}$ was the initial concentration of Ag^+^. The value of *R*_L_ was considered relevant with the degree of adsorption: it was unfavorable with *R*_L_ more than 1; it was favorable with 0 < *R*_L_ < 1; it was linear with *R*_L_ = 1 and it was irreversible with *R*_L_ = 0 [28]. In the paper, the calculated values of *R*_L_ for Ag^+^ onto TSC-CC resin were in range of 0.0177 to 0.0406 indicating that TSC-CC resin was suitable for the adsorption of Ag^+^.

### 3.5. Selective Adsorption

**Figure 7:**
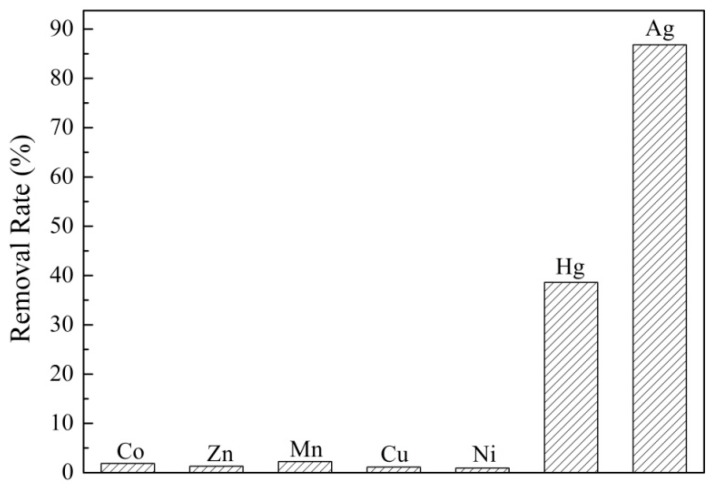
Selective adsorption of TSC-CC resin for Ag^+^.

Selective adsorption of Ag^+^ from acid solutions with coexisting ions (Hg^2+^, Ni^2+^, Co^2+^, Zn^2+^, Mn^2+^, and Cu^2+^) using the TSC-CC resin was investigated at acidity of 0.5 mol/L (Figure 7). The removal rate of Ag^+^ was higher than that of Co^2+^, Zn^2+^, Mn^2+^, Cu^2+^, and Ni^2+^. The removal rate of Hg^2+^ was about 35%. Because the thiourea groups in the structure of the resin have a positive effect for the adsorption of Hg^2+^ [29]. However, the Hg^2+^ was partly adsorbed and the removal rate of Hg^2+^ was lower than that of Ag^+^ (about 86%). The results indicated that the TSC-CC resin was a promising material for selective adsorption of Ag^+^ from the acid solutions with the coexisting ions.

### 3.6. Desorption and Recyclability of TSC-CC Resin

**Figure 8:**
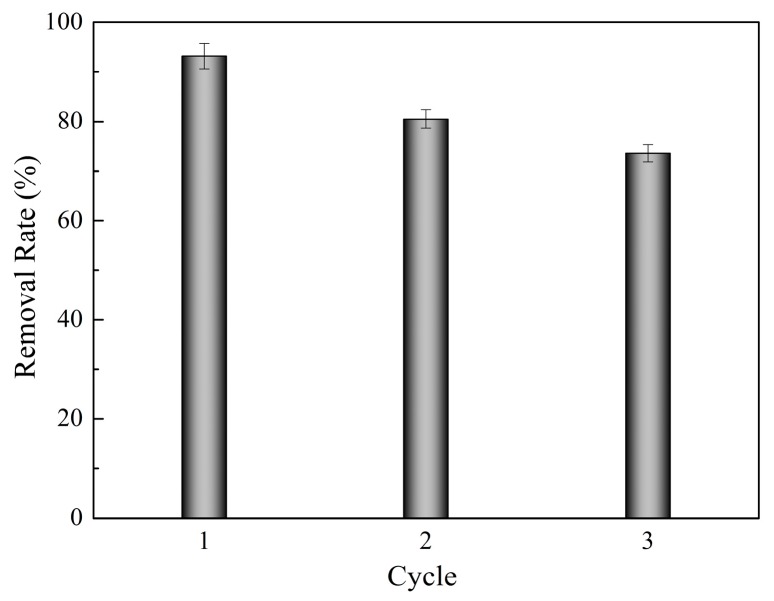
Recyclability of Ag^+^ on TSC-CC resin after three cycles of adsorption/desorption.

HCl (0.5 mol/L) solutions were employed as the desorption agent to regenerate the resin sorbent. To investigate the recyclability of resin, the TSC-CC (40 mg) was added into 80 mL of Ag^+^ solution (initial concentration was 320 mg/L, acidity was 0.5 mol/L) and the mixture solution was shaken for 16 h. After adsorption, TSC-CC was separated from solution by centrifuge and the supernatant was collected and analyzed. Then the sediment was eluted with desorption agent for 24 h, washed it using distilled water three times. The removal rate of Ag^+^ after adsorption/desorption is shown in Figure 8. After three regeneration cycles, the removal rate of Ag^+^ decreased from 93.1% to 73.6%, which may be due to the loss of adsorbent during the desorption process. The recyclability indicated that the TSC-CC resin had a good stability and can be recycled as a promising agent for removal of Ag^+^.

### 3.7. Adsorption Mechanism

**Figure 9:**
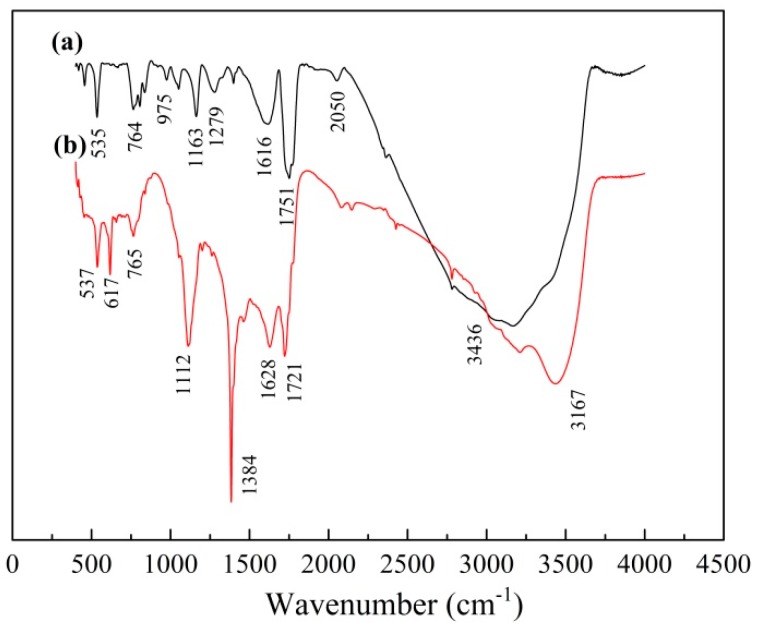
FT-IR spectra of (a) TSC-CC resin and (b) TSC-CC resin loaded Ag^+^.

Figure 9 presented the FT-IR spectra of TSC-CC resin before and after adsorption Ag^+^. Figure 9b presented a strong stretching vibration peak at 1384 cm^−1^. It was the stretching vibration peak of silver thiolates (Ag–S) in TSC-CC resin after adsorption Ag^+^ because of the interaction between the thiol group and Ag^+^ [16]. In addition, the peak at approximately 2050 cm^−1^ (–C=N^+^–H) may be caused by the thion-thioltautomerism of thioamide group –N–C=S in thiourea derivatives solution [2,30] (Figure 9a). The peak at 1279 cm^−1^ was corresponded to the –N–C=S of thiosemicarbazide moiety. Moreover, the stretching vibration peak of –N–C=S and C=N at 1616 and 1751 cm^−1^ moved to the 1628 and 1721 cm^−1^ after adsorption Ag^+^.

**Figure 10:**
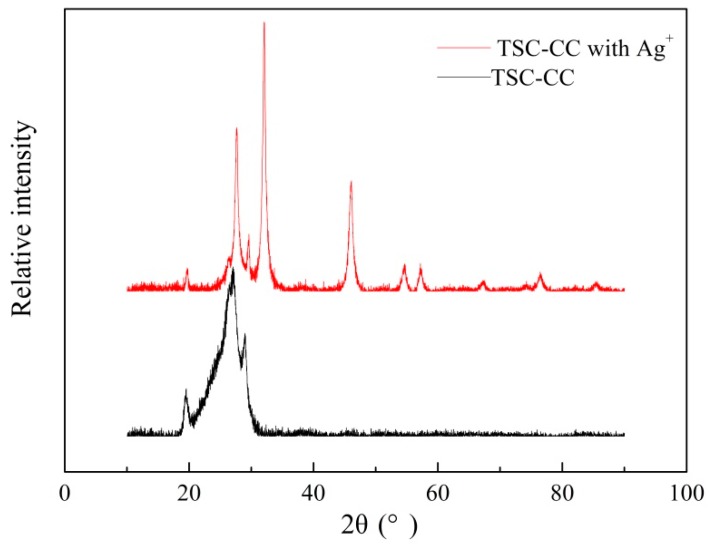
XRD curves of TSC-CC and TSC-CC with Ag^+^.

The XRD patterns of TSC-CC and TSC-CC with Ag^+^ were depicted in Figure 10. The XRD pattern of TSC-CC represents the distinct crystalline peaks at 19.5°, 27.1° and 28.8°. For TSC-CC with Ag^+^, the peaks width at 19.5°, 27.1° and 28.8° were narrowed, indicating the crystallinities were higher after adsorption Ag^+^. The increase of the crystallinities of TSC-CC with Ag^+^ was attributed to the reaction of sulfur and nitrogen functional groups with Ag^+^. In addition, the new peaks at about 32°, 45.9°, 54.3°, 57.2°, 67.3°, 76.4° and 85.4° further suggested the complexation reactions between the thiol group, amino, and Ag^+^ were conducted and generated the silver thiolates (Ag–S) and silver amide (Ag–N).

**Figure 11:**
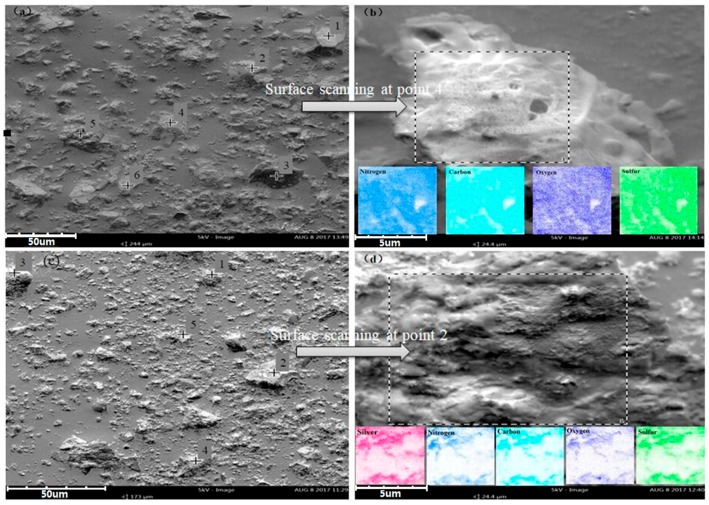
Scanning electron microscopy (SEM), images of TSC-CC resin (**a**,**b**); TSC-CC resin loaded Ag^+^ (**c**,**d**).

The morphology and element distribution of TSC-CC resins before and after adsorption Ag^+^ were investigated by scanning electron microscopy/energy dispersive spectrometer (SEM-EDS) (Figure 11). Figure 11a was the SEM images of the TSC-CC resin without adsorption Ag^+^, and several representative points were selected to analyze the element by point scanning, the point scanning results were presented in Table 4. In addition, the point 4 in Figure 11a was enlarged by surface scanning, the results are shown in Figure 11b and Table 4. It can be seen that the elements in chelating resin were evenly distributed and the nitrogen content of TSC-CC resin was about 60%, which can provide more functional groups so as to improve the adsorption capacity. Moreover, the presence of sulfur was beneficial to the selective adsorption of Ag^+^. The Figure 11c was the SEM images of the TSC-CC resin loaded Ag^+^, and the scanning results were listed in Figure 11d and Table 5. It showed that the silver content was high in TSC-CC resin after adsorption Ag^+^ and the combination of silver with sulfur and nitrogen was observed well. The above results indicating that the chelating resin was a promising adsorbent and Ag^+^ can be adsorbed largel by TSC-CC resin.

**Figure 12:**
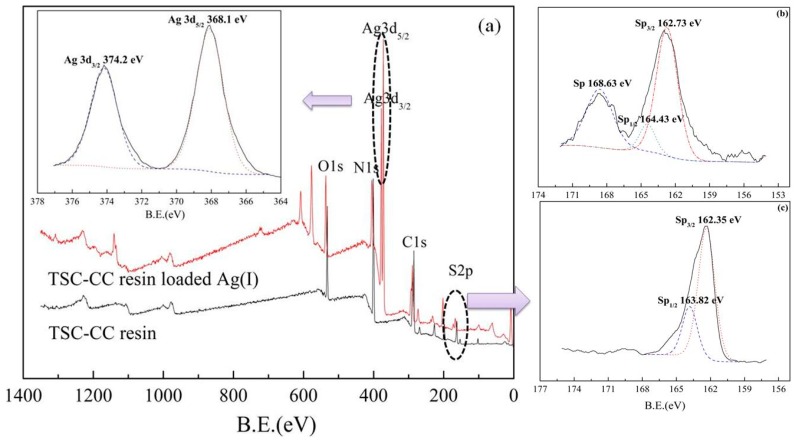
X-ray photoelectron spectroscopy (XPS) core-lever spectra of (**a**) TSC-CC resin before and after Ag^+^ adsorption; (**b**) S_2p_ after Ag^+^ adsorption; (**c**) S_2p_ before Ag^+^ adsorption.

To better evaluation of the adsorption mechanism, the resin before and after the adsorption of Ag^+^ was investigated by XPS. The wide-scan XPS spectra for the TSC-CC and TSC-CC loaded Ag^+^ were presented in Figure 12a. The predominant peaks were O_1s_, N_1s_, C_1s_, S_2p_, and Ag_3d_. It can be found that the new peak at 368.1 and 374.2 eV appeared after Ag^+^ adsorption. The presence of the nearby satellite band represented the Ag_3d_ orbital, and it confirmed that the Ag^+^ were adsorbed on the surface of the TSC-CC resin with a valance state of +1 [9].

The XPS spectra of S_2p_ were divided into two peaks at S_p1/2_ (162.35 eV) and S_p3/2_ (163.82 eV), attributed to the C=S bond in Figure 12c. However, after adsorption Ag^+^, the C=S bond shifted to S_p1/2_ (162.73 eV) and S_p3/2_ (164.43 eV), respectively, which indicating that a possible chemical change of S occurred on the surface and correspond to the sulfur bound to Ag^+^ [31]. It also can be found the peak of 164.43 eV was weak, which suggested that the group was protonated and attracted with Ag^+^ [2]. Moreover, a new peak was observed at 168.63 eV after adsorption Ag^+^ was assigned to the sulfur atoms in –SO*_x_* (contaminated S) [31].

**Scheme 2:**
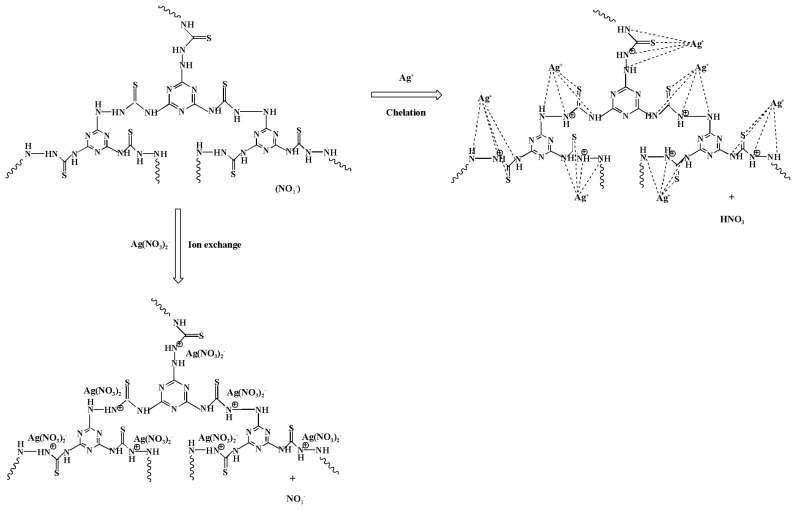
The adsorption mechanism of Ag^+^ on the TSC-CC resin adsorbent.

In the adsorption process of Ag^+^ by TSC-CC resin, the kinetics datas indicated that the adsorption rate was controlled by chemical reaction, which suggesting that the adsorption process was reliant on the amount of avaliable adsorption sites on the resin surface. It was ultimately controlled by the binding of Ag^+^ to the adsorbent surface or internal. In addition, the adsorption isotherm of TSC-CC resin fitted well by Langmuir isotherm model revealed that the adsorption process for Ag^+^ onto TSC-CC resin was homogeneous monolayer adsorption on the adsorbent surface. The main functional groups such as –N–C=S and –NH_2_ on the TSC-CC resin were involved in the coordination of Ag^+^. Therefore, the Ag^+^ adsorption by TSC-CC resin was a chemical adsorption with −N−C=S and $-{\mathrm{NH}}_{2}$ groups participated in the monolayer adsorption on the adsorbent surface and ionic reactions. The possible adsorption mechanism was proposed in Scheme 2.

## 4. Conclusions

The cyanuric-thiosemicarbazide chelating resin was successfully synthesized. The adsorbent was employed to selective recovery Ag^+^ from aqueous solutions. The results indicated that the optimal acidity was 0.5 mol/L and the maximum adsorption capacity of Ag^+^ was 872.63 mg/g. The adsorption isotherm was observed fitted well with the Langmuir isotherm model and the kinetic data preferably followed the pseudo-second order model for the chelating resin. FT-IR, XRD, SEM-EDS, and XPS indicated that the chelating and ionic interaction were mainly an adsorption mechanism. The high adsorption capacity and adapting strongly acidic condition suggested that the adsorbent presented a great potential to remove Ag^+^ from acid solutions. The recyclability indicated that the TSC-CC resin had a good stability and can be recycled as a promising agent for removal of Ag^+^.

## Figures and Tables

**Table 1:** Kinetics parameters of Ag^+^ adsorption on TSC-CC resin.

Kinetic Models	$q_{e}$ (mg/g) Calculated	Rate Constant	*R*^2^
Pseudo-first-order	581.18	$k_{1}=$ 0.1229 (min^−^^1^)	0.5181
Pseudo-second-order	699.3	$k_{2}=$ 0.000087 (g/mg min)	0.9982
Intraparticle diffusion	−	$k_{3}=$ 12.799 (mg/g min^0.5^)	0.8863

**Table 2:** Comparison of adsorption capacity for Ag^+^ with adsorbents in literatures.

Adsorbent	Adsorption Capacity (mg/g)	References
Chitosan resin	122.04	[24]
SE-SNPs	21.9	[7]
Chelating resin	313.2	[25]
Amino/thiol-bearing resin	308.88	[10]
Ag-IISHPs	80.5	[22]
TSC-CC resin	872.63	This study

**Table 3:** Isotherm parameters of Ag^+^ adsorption onto TSC-CC resin at 298 K.

Isotherm Model	Parameter	Value
Langmuir	$q_{m}$ (mg/g)	905.237
	$k_{L}$ (L/g)	0.0695
	*R*^2^	0.9742
Freundlich	$k_{F}$ (mg·g^−^^1^ (L·mg^−^^1^)^1/n^)	387.021
	1/n	0.1438
	*R*^2^	0.9672
Temkin	$k_{T}$ (L/g)	12.982
	$B_{T}$ (J/mol)	106.121
	*R*^2^	0.971

**Table 4:** Ultimate analysis of TSC-CC resin from energy dispersive spectrometer (EDS).

Number	Ultimate Analysis (%)			
	N	C	O	S
1	60.84	27.63	9.06	2.47
2	60.58	27.38	9.09	2.95
3	45.16	35.73	15.04	4.08
4	59.91	27.17	7.97	4.88
5	46.60	36.12	11.32	5.96
6	54.68	30.50	7.16	7.66
Surface scanning	58.92	27.48	9.23	4.38

**Table 5:** Ultimate analysis of TSC-CC resin loaded Ag^+^ from EDS.

Number	Ultimate Analysis (%)				
	Ag	N	C	O	S
1	46.53	15.22	28.42	9.83	−
2	29.30	28.53	27.46	11.7	2.93
3	49.36	13.80	25.85	8.28	2.70
4	41.40	16.14	22.62	16.25	3.52
5	44.04	17.49	28.19	8.39	1.89
Surface scanning	46.18	12.47	24.88	11.84	4.64

## References

[1] Kim Y.J., Choi J.H. (2012). Selective removal of nitrate ion using a novel composite carbon electrode in capacitive deionization. Water Res..

[2] Ying X., Li W., Jing X., Wang Y., Xing Z., Shan W., Lou Z. (2016). Selective recovery of Ag(I) coordination anion from simulate nickel electrolyte using corn stalk based adsorbent modified by ammonia-thiosemicarbazide. J. Hazard. Mater..

[3] Petrova Y.S., Pestov A.V., Usoltseva M.K., Neudachina L.K. (2015). Selective adsorption of silver(I) ions over copper(II) ions on a sulfoethyl derivative of chitosan. J. Hazard. Mater..

[4] Wang J., Huang C.P., Pirestani D. (2003). Interactions of silver with wastewater constituents. Water Res..

[5] Jeon C. (2017). Adsorption of silver ions from industrial wastewater using waste coffee grounds. Korean J. Chem. Eng..

[6] Liu C., Bai R., San Ly Q. (2008). Selective removal of copper and lead ions by diethylenetriamine-functionalized adsorbent: Behaviors and mechanisms. Water Res..

[7] Zhang L., Zhang G., Wang S., Peng J., Cui W. (2017). Sulfoethyl functionalized silica nanoparticle as an adsorbent to selectively adsorb silver ions from aqueous solutions. J. Taiwan Inst. Chem. Eng..

[8] Song X., Gunawan P., Jiang R., Leong S.S., Wang K., Xu R. (2011). Surface activated carbon nanospheres for fast adsorption of silver ions from aqueous solutions. J. Hazard. Mater..

[9] Wang S., Li H., Chen X., Yang M., Qi Y. (2012). Selective adsorption of silver ions from aqueous solution using polystyrene-supported trimercaptotriazine resin. J. Environ. Sci..

[10] Atia A.A., Donia A.M., Yousif A.M. (2005). Comparative study of the recovery of silver(I) from aqueous solutions with different chelating resins derived from glycidyl methacrylate. J. Appl. Polym. Sci..

[11] Yirikoglu H., Gülfen M. (2008). Separation and Recovery of Silver(I) Ions from Base Metal Ions by Melamine-formaldehyde-thiourea (MFT) Chelating Resin. Sep. Sci. Technol..

[12] Xue C., Zhu H., Zhang T., Cao D., Hu Z. (2011). Synthesis and properties of novel alkylbetaine zwitterionic gemini surfactants derived from cyanuric chloride. Colloids Surf. A.

[13] Zhang L., Ni C., Zhu C., Jiang X., Liu Y., Huang B. (2009). Preparation and adsorption properties of chelating resins from thiosemicarbazide and formaldehyde. J. Appl. Polym. Sci..

[14] Celik Z., Gülfen M., Aydin A.O. (2010). Synthesis of a novel dithiooxamide-formaldehyde resin and its application to the adsorption and separation of silver ions. J. Hazard. Mater..

[15] Juang R.S., Shao H.J. (2002). Effect of pH on competitive adsorption of Cu(II), Ni(II), and Zn(II) from water onto chitosan beads. Adsorption.

[16] Fu L., Zhang L., Wang S., Peng J., Zhang G. (2017). Selective adsorption of Ag+ by silica nanoparticles modified with 3-Amino-5-mercapto-1,2,4-triazole from aqueous solutions. J. Mol. Liq..

[17] Gülfen M., Kirci S., Aydin A.O. (2009). Separation and Recovery of Silver(I) Ions from Base Metal Ions by Thiourea- or Urea-Formaldehyde Chelating Resin. Sep. Sci. Technol..

[18] Birinci E., Gülfen M., Aydın A.O. (2009). Separation and recovery of palladium(II) from base metal ions by melamine–formaldehyde–thiourea (MFT) chelating resin. Hydrometallurgy.

[19] Febrianto J., Kosasih A.N., Sunarso J., Ju Y.H., Indraswati N., Ismadji S. (2009). Equilibrium and kinetic studies in adsorption of heavy metals using biosorbent: A summary of recent studies. J. Hazard. Mater..

[20] Liao B., Sun W.Y., Guo N., Ding S.L., Su S.J. (2016). Comparison of Co(^2+^) adsorption by chitosan and its triethylene-tetramine derivative: Performance and mechanism. Carbohydr. Polym..

[21] Qi C., Liu X., Lin C., Zhang X., Ma J., Tan H., Ye W. (2014). Degradation of sulfamethoxazole by microwave-activated persulfate: Kinetics, mechanism and acute toxicity. Chem. Eng. J..

[22] Hou H., Yu D., Hu G. (2015). Preparation and Properties of Ion-Imprinted Hollow Particles for the Selective Adsorption of Silver Ions. Langmuir.

[23] Kuang S.P., Wang Z.Z., Liu J., Wu Z.C. (2013). Preparation of triethylene-tetramine grafted magnetic chitosan for adsorption of Pb(II) ion from aqueous solutions. J. Hazard. Mater..

[24] Elwakeel K.Z., El-Sayed G.O., Darweesh R.S. (2013). Fast and selective removal of silver(I) from aqueous media by modified chitosan resins. Int. J. Miner. Process..

[25] Donia A.M., Atia A.A., El-Boraey H.A., Mabrouk D.H. (2006). Adsorption of Ag(I) on glycidyl methacrylate/*N*,*N*′-methylene bis-acrylamide chelating resins with embedded iron oxide. Sep. Purif. Technol..

[26] Cheng S., Zhang L., Xia H., Peng J., Shu J. (2017). Adsorption behavior of methylene blue onto waste-derived adsorbent and exhaust gases recycling. RSC Adv..

[27] Tang X.H., Zhang X.M., Guo C.C., Zhou A.L. (2010). Adsorption of Pb^2+^ on Chitosan Cross-Linked with Triethylene-Tetramine. Chem. Eng. Technol..

[28] Cheng S., Zhang L., Xia H., Peng J., Shu J., Li C. (2016). Ultrasound and microwave-assisted preparation of Fe-activated carbon as an effective low-cost adsorbent for dyes wastewater treatment. RSC Adv..

[29] Donia A.M., Atia A.A., Elwakeel K.Z. (2008). Selective separation of mercury(II) using magnetic chitosan resin modified with Schiff’s base derived from thiourea and glutaraldehyde. J. Hazard. Mater..

[30] Monier M., Kenawy I.M., Hashem M.A. (2014). Synthesis and characterization of selective thiourea modified Hg(II) ion-imprinted cellulosic cotton fibers. Carbohydr. Polym..

[31] Zhang M., Zhang Y., Helleur R. (2015). Selective adsorption of Ag+ by ion-imprinted *O*-carboxymethyl chitosan beads grafted with thiourea-glutaraldehyde. Chem. Eng. J..

